# Investigation of the Impact of L-Phenylalanine and L-Tyrosine Pre-Treatment on the Uptake of 4-Borono-L-Phenylalanine in Cancerous and Normal Cells Using an Analytical Approach Based on SC-ICP-MS

**DOI:** 10.3390/molecules28186552

**Published:** 2023-09-10

**Authors:** Emilia Balcer, Joanna Giebułtowicz, Małgorzata Sochacka, Anna Ruszczyńska, Magdalena Muszyńska, Ewa Bulska

**Affiliations:** 1 Radiochemistry Team, Reactor Research Division, Nuclear Facilities Operations Department, National Centre for Nuclear Research, Sołtana 7, Świerk, 05-400 Otwock, Poland; emilia.balcer@ncbj.gov.pl; 2 Department of Drug Chemistry, Pharmaceutical and Biomedical Analysis, Faculty of Pharmacy, Medical University of Warsaw, Banacha 1, 02-097 Warsaw, Poland; malgorzata.bogucka@wum.edu.pl; 3 Faculty of Chemistry, Biological and Chemical Research Centre, University of Warsaw, Żwirki i Wigury 101, 02-089 Warsaw, Poland; aruszcz@chem.uw.edu.pl (A.R.); magdalena.muszynska@pepolska.pl (M.M.); ebulska@chem.uw.edu.pl (E.B.); 4 Pro-Environment Polska Sp. z o.o., Żwirki i Wigury 101, 02-089 Warsaw, Poland

**Keywords:** 4-borono-L-phenylalanine, boron, boron neutron capture therapy, L-phenylalanine, L-tyrosine, single cell ICP-MS

## Abstract

Boron has gained significant attention in medical research due to its B-10 isotope’s high cross section for the reaction with thermal neutrons, generating ionizing particles that can eliminate cancer cells, propelling the development of boron neutron capture therapy (BNCT) for cancer treatment. The compound 4-borono-L-phenylalanine (BPA) has exhibited potential in BNCT clinical trials. Enhancing BPA uptake in cells involves proposing L-amino acid preloading. This study introduces a novel analytical strategy utilizing ICP-MS and single cell ICP-MS (SC-ICP-MS) to assess the effectiveness of L-tyrosine and L-phenylalanine preloading on human non-small cell lung carcinoma (A549) and normal Chinese hamster lung fibroblast (V79-4) models, an unexplored context. ICP-MS outcomes indicated that L-tyrosine and L-phenylalanine pre-treatment increased BPA uptake in V79-4 cells by 2.04 ± 0.74-fold (*p* = 0.000066) and 1.46 ± 0.06-fold (*p* = 0.000016), respectively. Conversely, A549 cells manifested heightened BPA uptake solely with L-tyrosine preloading, with a factor of 1.24 ± 0.47 (*p* = 0.028). BPA uptake remained higher in A549 compared to V79-4 regardless of preloading. SC-ICP-MS measurements showcased noteworthy boron content heterogeneity within A549 cells, signifying diverse responses to BPA exposure, including a subset with notably high BPA uptake. This study underscores SC-ICP-MS’s utility in precise cellular boron quantification, validating cellular BPA uptake’s heterogeneity.

## 1. Introduction

Cancer is a complex and life-threatening disease characterized by uncontrolled cellular growth and division. Developing effective cancer treatment is of utmost importance, prompting researchers to explore innovative approaches to enhance the effectiveness of cancer therapies. One such approach involves utilizing boron as a cell pre-treatment strategy. Boron has unique properties that have attracted attention in nuclear physics and medicine due to its B-10 isotope’s high cross section for the reaction with thermal neutrons, producing heavy ions such as charged lithium nuclei and alpha particles [1]. This specific reaction, involving boron and thermal neutrons, has led to the development of a novel cancer treatment method known as boron neutron capture therapy (BNCT). Although BNCT has primarily been applied in the treatment of head and neck cancers and melanoma, promising results suggest potential applications beyond these specific cancer types [1].

In clinical trials, a derivative of phenylalanine containing one boron atom per molecule, 4-borono-L-phenylalanine (BPA), has been extensively studied [1]. The transportation of BPA to tumor cells is governed by the active mode of L-amino acid transporters, particularly L-amino acid transporter-1 (LAT-1) [2]. These specific transporters are often overexpressed in various types of cancers due to abnormal tissue growth, leading to an increased supply of amino acids [3]. Consequently, BPA may be recommended for use in BNCT for different cancer types exhibiting overexpression of LAT-1 [4].

The evaluation of new applications of BPA should begin with in vitro studies measuring boron uptake in corresponding cell lines. A significant challenge in using BPA in BNCT is its short retention time in tumors [5] due to the antiport mechanism [6]. To enhance BPA uptake, preloading with other L-type amino acids, such as L-DOPA, L-tyrosine, and L-phenylalanine, can be considered [7,8]. However, the results vary depending on the specific cell line or harvested tissue. In the case of L-DOPA and L-tyrosine, pre-treatment either increased boron concentration [8,9,10,11] or showed no significant changes [7,12,13,14,15], while preloading with L-phenylalanine was reported to inhibit boron uptake [7,16]. To the best of our knowledge, there has been no study on L-amino acid preloading on normal (non-cancerous) cell lines thus far, except for the investigation of L-DOPA pre-treatment on the brain-around-tumor tissue harvested from the biopsy of a high-grade glioma patient [14].

BPA has been evaluated for use in BNCT in various cancer models, including glioblastoma multiforme, head and neck cancer, liver cancer, and lung cancer [1]. The initial research on BNCT and BPA for the treatment of non-small cell lung cancer (NSCLC) emerged in 2014 [17], providing a promising alternative to conventional radiotherapy, which often leads to serious complications [18]. However, BPA has only been tested on the human NSCLC A549 cell line [19,20,21,22,23,24] as a reference to novel boron carriers, and the effect of preloading with L-type amino acids in the case of lung cancer remains uninvestigated.

It should be stressed that it is crucial to select the appropriate method to analyze the cellular uptake of BPA. Previous studies on boron concentration in cells for BNCT were performed by mass or atomic spectrometry techniques such as inductively coupled plasma mass spectrometry (ICP-MS) [20,22], inductively coupled plasma atomic emission spectrometry (ICP-AES) [25], direct current plasma AES [26], flow-injection electrospray tandem mass spectrometry [27], and secondary ion mass spectrometry [14]. Some reports have also mentioned indirect methods based on alpha particle dosimetry [28]. The most clinically relevant methods of describing biodistribution are magnetic resonance imaging [29] and positron emission tomography, in the case of using F-18 labelled BPA (although it has to be underlined that while similar in structure, it is not chemically identical to BPA) [30]. However, these methods lack the spatial resolution to describe the cellular and subcellular distribution of BPA.

The determination of trace and ultra-trace boron concentrations can be easily performed by ICP-MS, which offers excellent sensitivity and low limits of quantification [31,32]. However, certain challenges can arise, including memory effects and poor washout, which may affect the accuracy of measurements [33,34]. Additionally, C-12 can overlap with the total B-11 concentration in biological samples. Moreover, the biological sample has to be acid-digested or homogenized, and the final result is the average value obtained from a batch sample [35]. The digestion increases the risk of sample loss or contamination, and other errors may occur due to the presence of dead cells. Furthermore, standard ICP-MS measurements commonly assume that each cell accumulates an equal amount of the analyzed element, resulting in the loss of information regarding differentiation within the cell population [36]. In cancer studies, where tumor heterogeneity plays a significant role, obtaining detailed knowledge about drug uptake is essential to understand the molecular mechanisms underlying the investigated therapy [37].

A novel alternative to address these limitations is single cell ICP-MS (SC-ICP-MS). With SC-ICP-MS, individual cells are directly introduced into the plasma for analysis. Each cell reaching the plasma generates a current spike, with the frequency corresponding to the number of cells and the intensity proportional to the concentration of analyzed elements [38]. Thus, SC-ICP-MS enables the investigation of variations in elemental uptake within a cell population, providing information about cell-to-cell variance, which is not available in standard ICP-MS measurements [39]. What is noteworthy is the possibility of employing SC-ICP-MS to also study the subcellular uptake of elements, which has already been described in the research investigating cis-platin uptake in cell nuclei [39].

While SC-ICP-MS has been applied in various fields, including drug development, cell biology, environmental studies, and metallomics, the analysis of boron has not been reported so far. A number of elements were tested by SC-ICP-MS, including Fe, S, Si, Zn, Pt, Ti, Gd, Au, Ag, As, Mn, Mg, Co, Nd, Eu, Tb, Dy, Ho, Tm, Yb, Lu, and Ir, as reviewed in [40,41].

The studies aimed to provide an approach based on ICP-MS and SC-ICP-MS for determining cellular boron concentration. This approach was employed to investigate the impact of L-phenylalanine and L-tyrosine pre-treatment on the uptake of BPA in both cancerous (NSCLC) and normal cells (lung fibroblasts). The use of SC-ICP-MS in boron analysis holds promise for expanding our understanding of cellular uptake and distributions of boron.

## 2. Results and Discussion

### 2.1. Influence of L-Phenylalanine and L-Tyrosine Pre-Treatment on Boron Concentration

**Figure 1:**
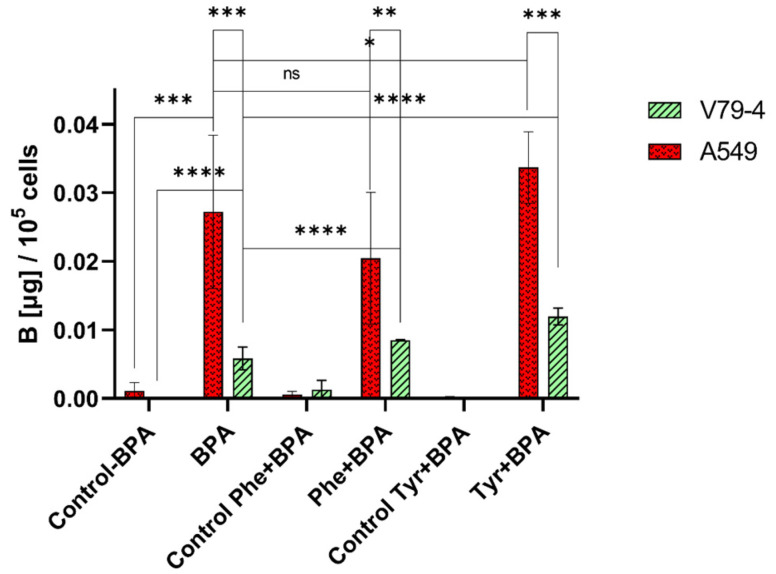
Boron accumulation in A549 and V79-4 cells after exposure to BPA and the influence of preloading with L-phenylalanine (Phe) and L-tyrosine (Tyr) on BPA uptake. Boron concentration is expressed as B [µg]/10^5^ cells. Data are given as mean ± SD from three independent experiments. Significance level symbols represent the results of unpaired *t*-tests between the studied variants: ns (not significant, *p* > 0.05), * (*p*  ≤  0.05), ** (*p*  ≤  0.01), *** (*p*  ≤  0.001), and **** (*p*  ≤  0.0001).

As discussed in the previous section, L-type amino acid pre-treatment of cells may increase the uptake of BPA. To further explore the influence of preloading with L-phenylalanine and L-tyrosine, experiments on A549 and V79-4 cell lines were conducted. The results, represented as the concentration of boron in batch cell samples obtained with the use of conventional ICP-MS, are summarized in Figure 1.

In the case of A549 and L-tyrosine pre-treatment, our results correspond with previous literature findings, also describing an enhancement of BPA uptake in other cancerous cell lines. We observed a statistically significant stimulating effect, increasing BPA uptake in cells 1.24 ± 0.47-fold (*p* = 0.028, 5 mM L-tyrosine solution). A similar study investigating the influence of L-tyrosine preloading on the uptake of the BPA analogue, 4-borono-2-[F-18]fluoro-L-phenylalanine, in human hepatocellular carcinoma HuH-7, human colorectal adenocarcinoma CaCo-2, and mouse melanoma B16-F1 cell lines, reported respective increases of boron uptake of 1.34 ± 0.57-fold (2.5 mM L-tyrosine solution), 1.04 ± 0.17-fold (1 mM L-tyrosine solution), and 1.57 ± 0.06-fold (2.5 mM L-tyrosine solution), after 60 min of preloading [11]. Some earlier reports also demonstrated nearly two-fold [8] and three-fold [10] increases in BPA uptake after L-tyrosine preloading in rat 9L gliosarcoma cells and B16-F1 mouse melanoma cells, respectively. Since A549 is a cancerous cell line, the amino acid supply is increased and LAT-1 is found to be over-expressed [42]. As described by Aldossari et al. [14], L-tyrosine preloading may stimulate the exchange and transport between the extracellular BPA and intracellular L-tyrosine, which is consistent with our observations. On the other hand, pre-treatment of A549 cells with L-phenylalanine seems to inhibit the uptake of BPA by a factor of 0.75 ± 0.86, but this result is not statistically insignificant (*p* = 0.068) and thus cannot be confirmed by this study. Previous literature reports described L-phenylalanine as a system L-specific agonist [7] and suggested that a low phenylalanine diet before the beginning of BNCT treatment may be advantageous for patients [43]. However, the inhibiting effect may also be beneficial due to the reduced radiation dose to the normal brain [16]. Nonetheless, as shown by the results of our study, pre-treatment of normal cells with L-phenylalanine may increase BPA uptake, so the dietary recommendation should be evaluated separately for different cancer types.

Similarly, in the case of the normal cell line V79-4, both L-phenylalanine and L-tyrosine pre-treatment exhibited an enhancing effect, resulting in factors of 1.46 ± 0.06 (*p* = 0.000016) and 2.04 ± 0.74 (*p* = 0.000066), respectively. It is most likely due to the increased intracellular accumulation of these L-amino acids, which stimulates the exchange and transport of extracellular BPA. The higher factors in the observed effect compared to the A549 cell line result from a very low uptake of BPA in V79-4 cells without the pre-treatment. This indicates that while normal cells do not exhibit an increased expression of LAT-1, the trans-stimulation of L-amino acids may have a similar effect, as in cancerous cells.

### 2.2. Boron Concentration in Individual Cells by SC-ICP-MS

**Figure 2:**
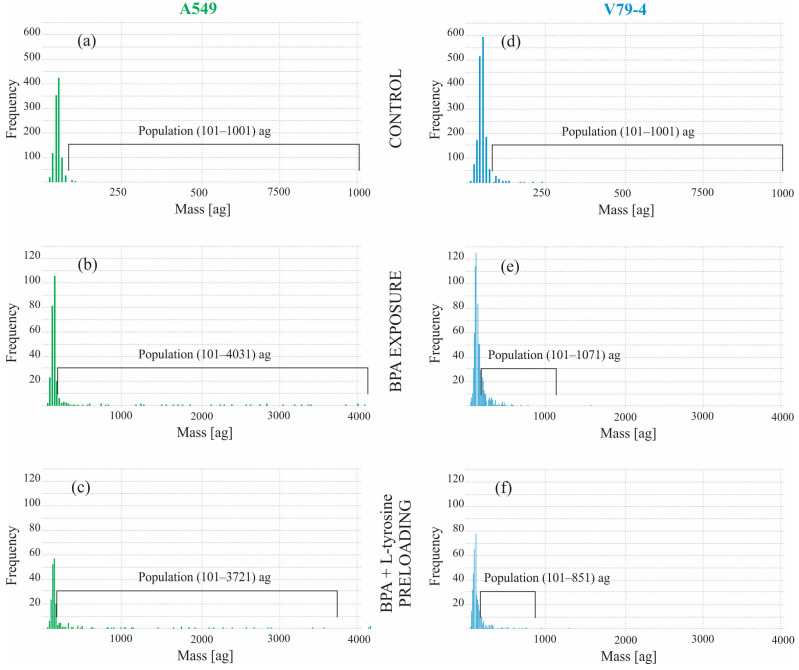
The histograms of the number of detected cells for the measured mass of boron in various conditions: (**a**) control group of A549 cell line, (**b**) BPA exposure of A549 cell line, (**c**) BPA + L-tyrosine preloading of A549 cell line, (**d**) control group of V79-4 cell line, (**e**) BPA exposure of V79-4 cell line, (**f**) BPA + L-tyrosine preloading of V79-4 cell line. A chosen population of cells above the baseline of 101 ag is highlighted in each histogram. The 5% of the highest mass signals corresponds to cell aggregates and was subtracted for each sample.

Based on the results obtained in batch cell samples conducted with conventional ICP-MS measurements, further studies on the preloading effect using SC-ICP-MS were performed using L-tyrosine pre-treatment. The results for the A549 and V79-4 cell lines after exposure to BPA and preloading with L-tyrosine are presented in Figure 2a–f in the form of histograms.

The most distinct observation during the analysis of histograms is the clear diversity in each sample, which corresponds with the genetic heterogeneity of cells, especially in the cancerous cell line. It has been established that cell lines originating from cancer tissues are subjected to transformations such as multiplication, mutation, and chromosomal rearrangement, resulting in the phenotypical and morphological variability within isolated cell lines [44]. That is the case of the nonhomogeneous A549 cell line, which consists of multiple clones [37], as reflected in the presented histograms that show a different cellular response to BPA within the tested population. The results further indicate that even though there is a notable difference in the uptake of BPA in A549 cells compared to V79-4 cells, as described in Section 2.1, only a part of the whole population of cells responds to BPA exposure, and the response varies. After the initial common peak for all samples, there is a visible difference between them after the threshold of 101 ag. Hence this value was chosen as a baseline to compare the separated population of cells, and the results for this highlighted population are summarized in Table 1.

In the case of the A549 samples, the highlighted population represents 39.45% of all detected cells in the BPA exposure group and 40.97% in the BPA exposure with L-tyrosine preloading group. Similarly, in the V79-4 samples, this population constitutes 38.72% of all detected cells in the BPA exposure group and 41.11% in the BPA exposure with L-tyrosine preloading group. In the agreement with predictions, the highlighted population in the control group (exposure to D-fructose solution without BPA) for both cell lines exhibits almost no impulses and constitutes 1.01% (A549) and 2.94% (V79-4) of the entire sample. The less distinct differences between the A549 and V79-4 cell lines than in the case of conventional ICP-MS measurements conducted in the batch cell samples are most likely due to the aggregation of V79-4 cells, which despite meticulous preparation was not avoidable, causing the possible appearance of signals from more than single cells on the histograms.

**Table 1:** The SC-ICP-MS results for the highlighted population above the threshold of 101 ag.

	Percentage of the Highlighted Population in the Entire Sample [%]		Mean Mass [ag] *	
Cell line	A549	V79-4	A549	V79-4
Control	1.01	2.94	447	205
BPA exposure	39.45	38.72	719	165
BPA + L-tyrosine preloading	40.97	41.11	619	179

* The mean masses are calculated by the Syngistix™ (PerkinElmer, Waltham, MA, USA) Single Cell Software (Version 2.5) Module using built-in algorithms.

However, the difference is still visible when comparing the mean mass in the separated population, e.g., 719 ag for A549 cells after BPA exposure and 165 ag for V79-4 cells after BPA exposure. The obtained results provide the first direct observation of the heterogeneity of BPA uptake in both cancerous and normal cell lines and show that a number of A549 cells exhibit an exceptionally high BPA uptake. Based on the collected data, we can conclude that ICP-MS provides the preliminary data on the total content of boron, and SC-ICP-MS measurements provide in-depth information on the BPA uptake. The microscopic evaluation of cells has to be a vital step in order to provide correct data interpretation. Nonetheless, it would be beneficial to combine SC-ICP-MS with flow cytometry to ensure the lack of interferences from aggregates. However, currently, such combinations are not routinely available.

## 3. Materials and Methods

### 3.1. Cell Cultivation

The human NSCLC A549 (ATCC^®^ CCL-185™ cell line was provided by the Medical University of Warsaw, Department of Applied Toxicology, Faculty of Pharmacy. The Chinese hamster lung fibroblast V79-4 (ATCC^®^ CCL-93™) cell line was provided by the Department of Environmental Health Science, Faculty of Pharmacy, Medical University of Warsaw. The A549 cell line was cultured in Kaighn’s Modification of Ham’s F-12 Medium (F-12K, Gibco, Paisley, UK) supplemented with 10% of fetal bovine serum (FBS, Gibco, Paisley, UK), 0.1 mg mL^−1^ streptomycin (Gibco, Paisley, UK), and 100 IU mL^−1^ penicillin (Gibco, Paisley, UK). The V79-4 cell line was cultured in Dulbecco’s Modified Eagle Medium (DMEM, Gibco, Paisley, UK) supplemented with 10% FBS, 0.1 mg mL^−1^ streptomycin and 100 IU mL^−1^ penicillin. Cells were maintained in a humidified atmosphere of 5% CO_2_ in air and 37 °C.

### 3.2. Cell Pre-Treatment with L-Phenylalanine and L-Tyrosine

A549 and V79-4 cells were seeded in 6-well (10^4^ cells/well) plates and allowed to attach and grow for 48 h. After removing the culture medium, the cells were rinsed with warm phosphate-buffered saline (PBS, Gibco, Paisley, UK) and incubated with Hank’s balanced salt solution containing calcium and magnesium cations (HBSS, Gibco, Paisley, UK). Alternatively, the cells were incubated in HBSS solution containing 5 mM L-phenylalanine (Sigma Aldrich, Steinheim, Germany) or L-tyrosine (Sigma Aldrich, Steinheim, Germany) at 37 °C for 60 min. Since the aim of the study was to prove the applicability of the proposed novel analytical approach, the conditions of preloading and BPA exposure experiments (concentrations and exposure time of amino acids and BPA) were carefully chosen based on previously described studies investigating the preloading effect on other cancerous cell lines [11,12,15]. After the incubation, the solutions were discarded, and the cells were exposed to BPA (Sigma Aldrich, Steinheim, Germany) according to the procedure described in Section 3.3.

### 3.3. Cell Exposure to 4-Borono-L-Phenylalanine

A549 and V79-4 cells were seeded in 6-well cell culture plates and cultivated for 48 to 72 h until reaching 80–90% confluency. First, the growth medium was removed, and the cells were washed with warm PBS. Then, the cells were incubated in a 2 mM BPA solution in the form of the D-fructose (LOBA Chemie Vienna, Fischamend, Austria) complex (1 g mL^−1^ D-fructose) at 37 °C for 120 min. The control group was incubated in a 1 g mL^−1^ D-fructose solution.

### 3.4. Sample Preparation for ICP-MS and SC-ICP-MS Measurements

After the incubation, the culture medium was first discarded, and the cells were washed with warm PBS. To detach the cells, they were exposed to trypsin (Gibco, Paisley, UK) for 3 and 7 min (V79-4 and A549 cell lines), respectively. Once detached, the cells were diluted with a growth medium and centrifuged at 25 °C for 7 min at 1000 rpm for A549 and 800 rpm for V79-4 cells lines. Subsequently, the medium was removed, and the cells were washed again with PBS and centrifuged under the same conditions. After that, the cells were exposed to 2% formaldehyde solution (Sigma Aldrich, Steinheim, Germany) in PBS and incubated for 10 min at 37 °C. Following the incubation, the cells were centrifuged again using the previously mentioned conditions; the formaldehyde solution was removed, and the cells diluted in PBS were kept refrigerated until the ICP-MS and SC-ICP-MS measurements were performed.

For ICP-MS measurements of boron concentration in batch cell samples, the samples were dissolved in 65% HNO_3_ after 2 h of ultrasonic stirring at 50 °C. Transparent solutions were transferred to plastic tubes and filled with deionized water before ICP-MS analysis was conducted, as described in [32]. Since the entirety of each sample was treated with 65% HNO_3_ and then measured, any extracellular BPA that may have been present in the sample due to possible leakage from cells was also included in the obtained result.

Preparing single cell samples is crucial for SC-ICP-MS measurements, and the number of cells in suspension must be carefully controlled to ensure that only one cell enters the plasma during the event. Therefore, the samples with the cells were washed a minimum of four times with fresh PBS until no boron was detected in the background. The boron content in the PBS solution after washing was checked before conducting the cell measurements. Subsequently, the cells were diluted to a final concentration of approximately 10^5^ cells/mL using PBS. The cells were counted using a haemocytometer (Neubauer improved counting chamber, Fisher Scientific, Vienna, Austria) and a light microscope (Olympus, Tokyo, Japan) with the 40× objective and 2.5× binocular. The cells were measured directly without any additional dilution.

For transport efficiency measurements, colloidal gold nanoparticles LGCQC5050 (LGC Limited, Teddington, UK) and EQ Four Element Calibration Beads (FLUIDIGM, Singapore) solutions were used.

High purity 65% HNO_3_ (J. T. Baker, Phillipsburg, NJ, USA or Merck, Darmstadt, Germany) with a final concentration of 1%, NORMATOM 25% ammonia (VWR Chemicals, Gdańsk, Poland) at final concentration 0.25%, Triton X-100 (PerkinElmer, Waltham, MA, USA) at final concentration 0.05%, and Milli-Q water (18.2 MΩ.cm, Merck, Darmstadt, Germany) were used during sample preparation.

### 3.5. Instrumentation

In this study, two spectrometers by PerkinElmer (Waltham, MA, USA) were used: a NexION 300D with a conventional sample introduction system for boron concentration in batch cell samples and a NexION 2000 spectrometer with a software extension for single cell measurements for the concentration of boron in individual cells. Experimental parameters, which were used during the measurements, are presented in Table 2. The working conditions of the spectrometers were optimized daily for maximum sensitivity and stability. An external 5-point calibration was used to obtain quantitative results. The memory effect was minimized by performing washouts between runs and ensuring that the signal reached the baseline after each sample. To control any possible interferences resulting from the presence of C-12 in the sample matrix, two isotopes of boron were measured (B-11 and B-10) in conventional ICP-MS experiments, and all measurements were performed in triplicates.

In the case of SC-ICP-MS experiments, a high efficiency nebulizer (HEN NEB) was applied to operate at a lower sample flow rate, generating smaller droplets and maximizing the signal from a small sample volume. An Asperon^TM^ (PerkinElmer, Waltham, MA, USA) spray chamber was used to enhance the transfer of micron-sized objects into the ICP-MS, surpassing traditional introduction systems. Preventing the loss of cells as a result of sticking to the walls of the spray chamber cells was accomplished by the All Matrix Solution (AMS) (PerkinElmer, Waltham, MA, USA) and additional gas flow. The system was also equipped with a syringe-driven Single Cell Micro DX Autosampler (PerkinElmer, Waltham, MA, USA), facilitating the delivery of intact cells into the instrument at a 10 µL min^−1^ flow rate.

Analysis was performed using the Syngistix™ (PerkinElmer, Waltham, MA, USA) Single Cell Software (Version 2.5) Module that uses algorithms to calculate, among others, transport efficiency (TE), threshold (sum of the mean of each sample signal and 3 times standard deviation), and particle detection limit (PDL), determining the lowest concentration of an element detected in a given SC-ICP-MS measurement. One of the key parameters in SC-ICP-MS analyses is TE. To determine this parameter, a method based on a known number of particles in two standard solutions was employed: one containing beads and the other containing gold nanoparticles. The bead-containing standard facilitates a more accurate representation of cell size, whereas the nanoparticle standard has certified concentrations, ensuring traceability.

### 3.6. Statistics

The statistical analysis was performed to evaluate the significance of the results. The data are presented as the mean ± standard deviation (SD) of boron concentration in batch cell samples determined with conventional ICP-MS and are derived from three independent experiments (*n* = 9). Differences between concentrations were considered significant at *p* < 0.05. Statistical analyses were performed with Statistica 13.3 using unpaired *t*-tests, and GraphPad Prism 9.5.1 software was used for graphs.

## 4. Conclusions

In this work, we successfully employed the SC-ICP-MS technique to measure boron concentration in cell lines exposed to BPA, providing a novel method for determining boron content at the individual cell level and estimating the efficiency of the uptake of boron agents. Pre-treatment with L-tyrosine and L-phenylalanine demonstrated enhanced BPA uptake in the normal cell line. However, in the NSCLC cell line, the enhancement was only observed for L-tyrosine. Interestingly, the cancerous cell line exhibited significant heterogeneity in boron content. These results confirm the importance of dietary restriction regarding L-phenylalanine content due to its possible influence on BPA uptake in normal cells. For successful employment of SC-ICP-MS in boron uptake studies, the possibility of cell aggregate formation has to be carefully evaluated at the sample preparation stage, and the additional use of flow cytometry could be considered to overcome this issue.

## Figures and Tables

**Table 2:** The experimental parameters for ICP-MS and SC-ICP-MS instruments.

Parameter	ICP-MS	SC-ICP-MS
Instrument	NexION 300D (PerkinElmer)	NexION 2000 (PerkinElmer)
Mass analyzer	Quadrupole	Quadrupole
Measurement Mode	Standard	Standard
Nebulizer	Quartz Mainhardt	HEN Mainhardt
Spray chamber	Quartz cyclonic	Quartz Asperon
Carrier gas	Ar	Ar
Nebulizer gas flow	0.88 L min^−1^	0.40 L min^−1^
Sample introduction	Manually, peristaltic pump	SC Micro DX, syringe pump
Sample Flow Rate	0.2 mL min^−1^	10 μL min^−1^
Dwell time	100 ms	50 μs
AMS gas flow	n/a	0.7 L min^−1^
Measured isotopes *	B-10, B-11	B-10, B-11
Transport Efficiency	n/a	28%
Sample preparation	Digestion and dilution	Dilution with PBS to ~10^5^ cells mL^−1^
**Analytical Parameters**	**Digestion + ICP-MS**	**Dilution + SC-ICP-MS**
Calibration range	(10–100) μg L^−1^ in 1% HNO_3_	(1–5) μg L^−1^ in PBS
Regression equation **	y = 1526x + 157.13	y = 0.8108x + 0.335
Correlation coefficient, R^2^	0.9999	0.9999
LOD/PDL	20 ng kg^−1^	12 ag/cell
Recovery	100–115%	n/a
Bin size	n/a	10 ag
Threshold	n/a	1.06–3.05

* The signal monitoring for B-10 and B-11 was similar for both in the case of both methods. Therefore, it was decided to conduct measurements using B-11 due to its higher abundance and, consequently, better sensitivity. ** Regression equation of (i) ICP-MS measurements relates the dependence of intensity expressed in count per second (cps) to concentration (μg), and (ii) SC-ICP-MS relates the dependence of intensity expressed in counts to max flux (μg/event).

## Data Availability

The data presented in this study are available on request from corresponding author.
